# PI3-Kinase δγ Catalytic Isoforms Regulate the Th-17 Response in Tuberculosis

**DOI:** 10.3389/fimmu.2019.02583

**Published:** 2019-11-01

**Authors:** Gina R. Leisching

**Affiliations:** SA MRC Centre for TB Research, DST-NRF Centre of Excellence for Biomedical Tuberculosis Research, Division of Molecular Biology and Human Genetics, Faculty of Medicine and Health Sciences, Stellenbosch University, Cape Town, South Africa

**Keywords:** tuberculosis, IL-17A, PI3-Kinase, Th17, neutrophils, airway inflammation

## Abstract

Although IL17A plays a protective role at the mucosal surface, when IL17A signaling becomes dysregulated, a pathological response is locally induced. At the early stages of *Mycobacterium tuberculosis* (*M.tb*) infection, IL17A contributes to granuloma formation and pathogen containment. In contrast, during disease progression, a dysregulated IL17A hyperinflammatory response drives tissue destruction through enhanced neutrophil recruitment. Cumulative research has implicated the PI3-Kinase pathways as one of the most relevant in the pathophysiology of inflammation. Evidence shows that IL-17A secretion and the expansion of the Th17 population is dependant in PI3-Kinase signaling, with the p110δ and p110γ isoforms playing a prominent role. The p110γ isoform promotes disease progression through dampening of the Th17 response, preventing pathogen clearance and containment. The p110γ gene, *PIK3CG* is downregulated in TB patients during late-stage disease when compared to healthy controls, demonstrating an important modulatory role for this isoform during TB. Conversely, the p110δ isoform induces IL-17A release from pulmonary γδ T-cells, committed Th17 cells and promotes neutrophil recruitment to the lung. Inhibiting this isoform not only suppresses IL-17A secretion from Th17 cells, but it also inhibits cytokine production from multiple T-helper cell types. Since increased IL-17A levels are observed to be localized in the lung compartments (BAL and lymphocytes) in comparison to circulating levels, an inhalable PI3Kδ inhibitor, which is currently utilized for inflammatory airway diseases characterized by IL-17A over-secretion, may be a therapeutic option for active TB disease.

## Introduction

IL-17A is mostly active at mucosal sites and plays a primarily protective role at the lung surface through bridging the gap between the innate and adaptive immune responses (1, 2). Dysregulated signaling upstream or downstream may therefore greatly affect feedback loops during an inflammatory or infectious episode. Its potential of IL-17A to mediate a pathological immune response is especially observed at the intestinal, skin, and lung mucosa. Diseases such as colitis, inflammatory bowel disease (IBD), dermatitis, psoriasis, asthma, and chronic obstructive pulmonary disease (COPD) caused by chronic inflammation are rooted by dysregulated IL-17A signaling (3–7). Its role in potentiating chronic inflammatory diseases is so significant, that targeting of the IL-17A pathway is a major focus of anti-inflammatory drug development (8).

The immunopathological potential of IL-17A during autoimmune and infectious episodes suggests that IL-17A may have a detrimental effect in chronic bacterial infections such as tuberculosis (TB), particularly during late stages of disease (9). In resistant hosts, IL-17A contributes to the formation of a mature granuloma (10, 11) which constrains the multiplication of *Mycobacterium tuberculosis* (*M.tb*) clinical isolates and plays a protective role (12).This is particularly observed during infection with hypervirulent *M.tb* strains. A recent paper by Erdmann and colleagues describes the ability of IL-17A to contribute to the expansion of IFN-γ-, IL-2- and TNF-secreting multifunctional T cells in IL-27Rα^−/−^ mice, and that the level of IL-17A induction is essential for the accumulation of these cells during infection (13, 14). It was also observed that IL-17A, but not IFN-γ, is critical for mucosal vaccine–induced immunity against *M.tb* (14). On the other hand, Th17 cells have been implicated in the pathology of TB through inducing exacerbated neutrophil recruitment which enhances inflammation and pleural tissue damage in during disease (3). Even though IL-17 production may be required for early granuloma formation, it's chronic or exacerbated secretion may also be detrimental as it promotes neutrophil accumulation which compromises the generation of a stable mononuclear granuloma. Granuloma turnover then becomes dysregulated which leads to liquefactive necrosis and pathological scarring (15–17). Cumulative evidence suggests that IL-17A-induced pathology is significant during late-stage disease rather than during early infection (3, 18, 19), however delineating IL-17A signaling during both stages could promote the development of specific immunotherapeutic interventions that target key molecules within this pathway, and in doing so, improve current treatment regimes.

It has been observed that in patients with active TB, not only do they have a lower proportion of circulating Th17 cells (20), but plasma levels of IL-17A are also lower when compared to healthy individuals or those with latent TB infection (LTBI) (21). IL-17A is found to be increased in bronchoalveolar lavage (BAL) fluid of TB patients (17) and in lymphocytes surrounding pulmonary granulomas (17). In mice studies, it was observed that IL-17RA (IL-17A receptor) is expressed in non-hematopoietic cells (endothelial and epithelial cells, as well as fibroblasts) in the lungs of *M.tb* infected mice (18, 22). Thus, during late-stage disease, IL-17A appears to play a predominant role within the lung microenvironment and through complex signaling cascades, not only promotes neutrophil influx into the pleural space, thereby promoting the damaging hyperinflammatory environment, but simultaneously drives tissue destruction through upregulating matrix metalloproteases (MMPs) (17).

The literature indicates that various PI3-K isoforms play a significant role in shaping the Th17 response, however signaling through the various isoforms is still unclear, as well as the role they play in IL-17A secretion in TB (4, 23, 24). It is established that IL-17A secretion and the expansion of the Th17 population is dependent on IL-23 (16), however downstream signaling is mediated by members of the PI3-Kinase family. Studies have shown that the PI3-Kinase pathway activates and is activated by IL-17A during a number of inflammatory diseases (25, 26), including TB (17, 27). It is noteworthy that transcriptomic studies have revealed that various members of the PI3-kinase family, such as *PIK3CD, PIK3IP1, PIK3C2B* are aberrantly expressed in patients with active TB compared to uninfected controls (28, 29). This review aims to clarify PI3-Kinase-mediated IL-17A signaling in the context of TB disease in various cell types and indicate how it mediates the Th17 response. In addition, the possibility of utilizing PI3K-isoform specific drugs which could hinder Th17 differentiation, and therefore IL-17A secretion during late-stage TB disease as a possible therapeutic option is explored.

## PI3-kinases Regulate IL-17 Secretion and T-Cell Expansion

PI3-Kinases are fundamental intermediaries in immune cell signaling networks. They generate a phosphatidylinositol (3,4,5)-trisphosphate second messenger molecule which recruits protein kinases and other proteins to the plasma membrane, where they activate other downstream mediators that are important in cell differentiation, proliferation, migration, and survival. The class IA PI3Ks, namely PI3Kα, PI3Kβ, and PI3Kδ bind tyrosines phosphorylated by receptor-associated kinases, whereas PI3Kγ, the only class IB PI3K, is activated by G protein-coupled receptors (30). PI3Kδ and PI3Kγ are expressed predominantly in leukocytes and have been studied intensively in the context of immune-mediated diseases. Further, both isoforms are required in some cellular responses such as the generation of ROS by neutrophils and the degranulation of mast cells, which suggests that the contribution of each isoform is coordinated at different stages (31–33).

**Figure 1:**
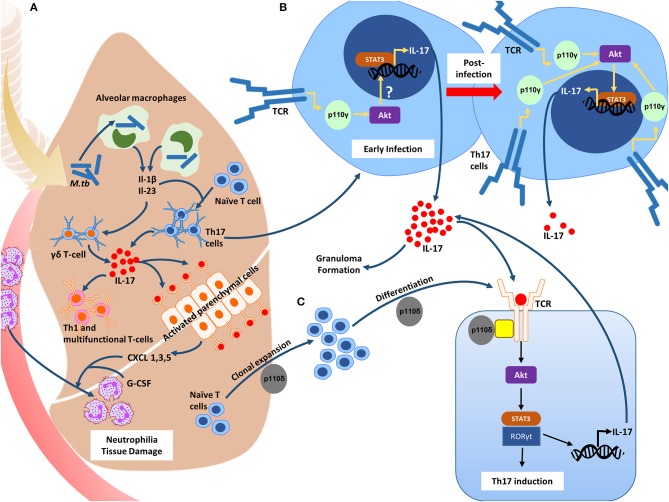
The role of the PI3-K δγ catalytic isoforms in inducing the Th17 phenotype during *M.tb* infection. **(A)** Inhaled mycobacteria in the lung is phagocytosed by resident alveolar macrophages, inducing the release of IL-1β and Il-23. These cytokines, as well as others cause the recruitment of circulating immune cells such as neutrophils and lung γδ T-cells. IL-17A is then released from the γδ T-cells causing the activation of the parenchymal cells which through the release of both CXCL1, 3, and 5, and G-CSF, promote an influx of neutrophils. IL-17A levels are essential for the recruitment of Th1 and multifunctional T-cells. **(B)** The PI3-K p110γ isoform is downregulated in activated Th17 cells during the initial stages of infection, and through an unknown downstream mechanism, results in enhanced *IL17A* transcription, and subsequent IL-17A release. In resistant hosts, this results in granuloma formation and effective containment of the bacilli. PI3-K p110γ expression returns to homeostatic levels, leading to the suppression of *IL17A* transcription. In susceptible hosts, PI3-K p110γ expression remains downregulated, resulting in increased IL-17A secretion and pathology. **(C)** The PI3-K p110δ isoform is required for the clonal expansion and differentiation of Th17 cells +.

The role of these isoforms in the context of the Th17 response in TB has been better defined in the last few years where the immunomodulatory role early on in infection is observed. Briefly, the presence of inhaled mycobacteria in the lung activates local bronchial epithelial cells (34) and phagocytosis by alveolar macrophages mediated by various receptors marks the commencement of the innate immune response during the early stages of infection (35). Fine orchestration of the release of chemokines and cytokines from these cells quickly recruits circulating immune cells such as neutrophils and lung γδ T-cells to the site of infection which is then followed by effector T-cell populations (Figure 1A). Naïve CD4^+^ T-cells differentiate into Th17 cells after exposure to IL-1β and IL-23 (36), which in turn stimulate the secretion of IL-17A. TB-reactive T-cells residing in the lungs before *M.tb* exposure and those located systemically are recruited to the site of infection shortly after pulmonary *M.tb* exposure, which is where the control of the initial infection begins (37). Activation of PI3-kinase signaling is initiated downstream of the T-cell receptor after its engagement with an antigen. The increment in T-cell populations and the related cytokines is a hallmark of the majority of immunological diseases, including TB. The significance of PI3-kinases in regulating the development, survival and differentiation of Th1, Th2, and Th17 cell subsets has been demonstrated.

### PI3-K p110γ Inhibits IL-17A Secretion During TB

PI3Kγ, a class IB isoform belonging to the PI3-Kinase family plays a prominent role in shaping the Th17 response during early infection with *M.tb* (Figure 1B). Studies in gene-deficient mice suggest that the presence of the p110γ isoform may promote susceptibility to infection and subsequent development of TB disease through dampening of the Th17 response (38–40). It was observed that PI3Kγ^−/−^ mice challenged intranasally with LPS had increased concentrations of IL-17A in their bronchoalveolar lavage fluid (39). Another group showed that spleen cell cultures stimulated with LPS revealed increased production of IL-17A by PI3Kγ- deficient T-cells (40). More recently, Cavalcanti-Neto et al. observed that mice deficient in this enzyme were resistant to *M.tb* infection and exhibited a robust Th17 response to which they attributed the improved resistance (38). In line with this, transcriptomics reveal that in patients with active TB, *PIK3CG* is downregulated compared to healthy controls (41), and those with latent TB (42) indicating a modulatory role for this isoform even during late stage disease. This data suggests that in resistant hosts, PI3Kγ may be downregulated initially in order to facilitate granuloma formation and resolution of the infection through IL-17A upregulation, and then regain homeostatic levels of expression after the resolution of the infection. In hosts which are susceptible to progress toward active disease, PI3Kγ may be downregulated from the initial point of infection throughout the progression and establishment of disease resulting in the over secretion and therefore pathological potential of IL-17A. This dysregulated expression may be as a result of inherited genetic alterations in *PIK3CG* and/or dysregulation in the upstream or downstream signaling components which govern PI3Kγ activation. This requires further investigation. Nevertheless, lower levels of the p110γ isoform likely contributes to the over-secretion and therefore the immunopathology of the IL-17A response during TB, and raises the question of whether PI3Kγ classifies as a negative regulator of IL-17A during natural protective immunity toward *M.tb* infection.

### PI3-K p110δ Is a Target of *M.tb* and Shapes the Th17 Response During TB

Pharmacological studies together with genetic evidence have implicated dysfunctional PI3K signaling in airway inflammation, and studies exploring the potential therapeutic benefit of PI3Kδ (class IA isoform) have yielded encouraging results (43). PI3Kδ is an isoform of PI3Kγ and lies downstream of tyrosine kinase-associated receptors, T cell receptors (TCR), co-stimulatory, and cytokine receptors, depending on the cell type. The role of PI3Kδ in macrophages and T-cells during *M.tb* infection is discussed below.

Recent work has found that during macrophage infection, *M.tb* upregulates miRNAs that target mRNAs encoding PI3Kδ, mTORC-1, and MNK-1 (44). By disrupting the genes encoding PI3-K δ/AKT/mTORC1 and MNK regulatory pathways, matrix metalloproteinase (MMP) expression, specifically MMP-1, is upregulated. In doing so, *M.tb* alters the normal protective response of the macrophage toward a tissue destructive phenotype through MMP expression. In the same study the authors observed that the expression of PI3Kδ is globally absent throughout TB granulomas, but present in normal lung tissue from healthy individuals (44). Knock-out studies found that p110γ/δ^−/−^ mice had increased IL-17A serum concentrations and frequencies of IL-17A+ splenic T-cells and that a deficiency in either the γ or δ isoform disrupts the IL-17A/G-CSF axis which results in neutrophilia (27). Evidence suggests that this decrease or loss in function of PI3Kδ enhances IL-17A and IL-17A-producing T-cells. In contrast, inhibition of PI3Kδ suppresses IL-17A expression through the regulation of NF-kB activity in a murine model of asthma (45) and in imiquimod-induced psoriasis-like dermatitis (6). Recently, a disease related to a gain of function mutation in PI3Kδ was described (46). In activated PI3Kδ syndrome (APDS), over expression of PI3Kδ in these individuals predisposes them to respiratory infections and airway damage. Thus, both a gain or loss in function of PI3Kδ significantly affects the Th17 response during an infectious or inflammatory episode and plays an important role in immunopathology of various disease states.

The signaling pathways responsible for T-cell differentiation are numerous, however the importance of the PI3-Kinase pathway in orchestrating differentiation of these cells has been noted previously (25, 26, 47–50). PI3Kδ specifically is of interest since its preferentially expressed in leukocytes and is essential for CD4^+^ T-cell clonal expansion and differentiation (51). Naïve cells differentiate into Th17 cells following stimulation of their T-cell receptor (TCR) by antigen exposure or to IL-1β and IL-23 stimulation (36). Upon stimulation, the TCR causes PI3-K δ activation through TCR costimulatory and adaptor proteins (51, 52) which then generates phosphatidylinositol-3,4,5-trisphosphate (PIP3) by phosphorylating phosphatidylinositol-4,5-bisphosphate at the plasma membrane. PIP3 then recruits AKT to the plasma membrane inducing its phosphorylation where it initiates various cellular functions. Recently it was observed that PI3Kδ induces IL-17A secretion from committed Th17 cells (Figure 1C), and that inhibiting this isoform not only suppresses IL-17A secretion from Th17 cells, but also inhibits cytokine production from multiple T-helper cell types (48). The same study confirmed that PI3Kδ mediated IL-17A release from pulmonary γδ T-cells, and thus appears to be a key molecule in the IL-17A signaling cascade. Inhibition of PI3-K δ *in vivo* also resulted in a decrease in neutrophil recruitment to the lung and therefore in the BAL fluid of mice. No effect was observed in macrophages and other cells which demonstrates the link between PI3Kδ, IL-17A, and neutrophil trafficking (48).

As a consequence of dysregulated IL-17A signaling resulting from changes in the expression of PI3Kδ or PI3Kγ, exacerbated neutrophil influx into the pleural space is commonly observed during late stage TB (53). Further, aberrant expression of these genes and other PI3-K family members cause disruptions in neutrophil trafficking that lead to neutrophil hyperreactivity through directly compromising their migratory accuracy. This results in prolonged tissue transit time which leads to bystander tissue injury mediated by surface-associated neutrophil proteases and further secretion of IL-17A (54). Targeting of the p110δ isoform that control neutrophil influx and IL-17A may be a therapeutic option for patients with TB.

## Is the Inhibition of p110δ Isoform an Option for the Treatment of TB?

The benefit of targeting PI3K isoforms has received considerable attention and is being viewed as a viable therapeutic option in inflammatory and infectious lung disorders. The comprehensive role of PI3Kδ and PI3Kγ in the ordinance of immunological mechanisms has presented them as potential targets for the treatment of immunological diseases such as autoimmune and allergic diseases (55, 56). Examples of this include PI3Kδ inhibitors to suppress the progression of inflammation and reduce the severity of rheumatoid arthritis and systemic lupus erythematosus in murine models (57). The selective PI3Kγ inhibitor AS605240 was shown *in vitro* and *in vivo* to reverse autoimmune diabetes in non-obese diabetic mice and inhibit T-cell cytokine release (58). In the context of TB, Th1 along with Th17 are the main effector populations which mediate both protection and pathology during the early and late stages of disease progression (9, 59). Thorough investigation of the functions of PI3Kδ and PI3Kγ in tuberculosis and other disease models, as well as in patient tissues, will be crucial to validate them as targets for the treatment of a range of inflammatory diseases. As mentioned previously, dysregulated IL-17A secretion is also observed to be increased in the airways of COPD patients and severe asthmatics which coordinate neutrophilic inflammation in these diseases. Although neutrophilia is an overwhelming feature of these diseases, targeting of these cells may not be effective. It was observed that therapies directed at neutrophil-derived products have limited efficacy and are only effective exceptional circumstances (60, 61). The p110δ isoform has been implicated in airway inflammation, with selective targeting of this isoform yielding promising results in COPD and asthma by broadly reducing lymphocyte-derived cytokines such as IL-17A, and suppressing ROS release from neutrophils (43, 62, 63). Specifically, p110δ inhibition demonstrates that T lymphocyte-derived cytokine generation can be suppressed with multiple T-cell lineages targeted (48), as well as Th17 differentiation (48). Recently, a clinical study on APDS patients was conducted using the oral PI3Kδ selective inhibitor Leniolisib which aimed to “normalize” the augmented PI3Kδ activity rather than to completely inhibit it. This resulted in some clinical benefit in a short-term study, without significant toxicity to the patient (64).

In TB patients it is imperative that IL-17A secretion is not completely abolished as this cytokine is required for a certain level of *M.tb* control and granuloma turn over. Further, an inhalable form of this inhibitor, for use in asthma patients may be a viable option. The above evidence suggests that targeting of this isoform may yield positive results. It is more likely that by dampening the Th17 response, which includes neutrophil influx, tissue damage will be reduced. In this way, long-term pulmonary damage from fibrotic scar tissue development may be subverted, however the resulting effect on *M.tb* survival is unclear and requires further investigation. Although IL-17A does exhibit protective effects, particularly against hypervirulent *M.tb* strains, the option of enhancing IL-17, particularly during late-stage TB disease may potentiate and exacerbate auto-immune responses and chronic inflammation. Further investigation into the Th17 response during late-stage TB is required to increase our understanding on the dynamic changes in IL17 signaling during this complex disease.

## Outstanding Questions and Challenges

An important step would begin with investigations in the lung and pulmonary compartment of TB patients as this will reveal unique characteristics of this niche which are distinct from what is observed in the blood. For example there is evidence supporting the fact that Th17-like cells and Th17- related cytokines are remarkably different from those observed from the blood in TB patients (64). Thus, better characterization of the Th17 cellular response in TB pulmonary compartments is needed. Since Th17 cells and their related cytokines are increased in pulmonary tissue and BAL during late-stage disease (17), an inhalable p110δ isoform inhibitor is a therapeutic treatment option as an adjunct to standard antibiotics. To date, the use of p110δ isoform inhibitors as an adjunct to standard therapy have not been proposed for the treatment of TB, thus many unanswered questions remain as to whether this is a viable option. Inflammatory airway diseases with an inflammatory cytokine profile similar to that of TB, which is driven specifically by IL-17A oversecretion, appear to be resolved with the use of a p110δ inhibitor such as Leniolisib. Extended studies are required to evaluate outcomes such as respiratory infection and inflammation, and to establish the safety of long-term treatment of the drug. Evidence suggests that PI3Kδ and PI3Kγ synchronize in distinct, yet interdependent signaling pathways in many immune cells. Their fine-tuned integration gives rise to pro-inflammatory events in the multistep pathogenic process of inflammation. To date, it is unknown how the expression of these isoforms are regulated in response to one another, as well as other isoforms, particularly during TB. A key question that remains is whether both PI3Kδ and PI3Kγ are valid targets for the treatment of chronic inflammatory diseases such as tuberculosis or whether one would be more suitable than the other. Further, the presence of *M.tb* in the lungs of TB patients receiving the proposed treatment raises further questions: (1) will a reduction in IL-17A levels provide a niche more suitable for *M.tb* replication, or (2) will it reduce neutrophil recruitment to the lung compartment, thereby promoting the generation of a stable mononuclear granuloma?

## Author Contributions

The author confirms being the sole contributor of this work and has approved it for publication.

### Conflict of Interest

The author declares that the research was conducted in the absence of any commercial or financial relationships that could be construed as a potential conflict of interest.
